# Smoking and Socio-Demographic Determinant of Cardiovascular Diseases among Males 45+ Years in Indonesia

**DOI:** 10.3390/ijerph8020528

**Published:** 2011-02-16

**Authors:** Wasis Sumartono, Anna M. Sirait, Maria Holy, Hasbullah Thabrany

**Affiliations:** 1 Center for Humanities, Health Policy and Community Empowerment, National Institute of Health Research and Development, Indonesian Ministry of Health, Jl. Percetakan Negara No. 23 A, Jakarta 10560, Indonesia; 2 Center for Health Intervention Technology, National Institute of Health Research and Development, Indonesian Ministry of Health, Jl. Percetakan Negara No. 29, Jakarta 10560, Indonesia; E-Mail: annamaria@litbang.depkes.go.id; 3 Center for Applied Health Technology and Clinical Epidemiology, National Institute of Health Research and Development, Indonesian Ministry of Health, Jl. Percetakan Negara No. 29, Jakarta 10560, Indonesia; E-Mail: maria@litbang.depkes.go.id; 4 Center for Health Economics and Policy, School of Public Health, University of Indonesia, Depok, West Java, Indonesia; E-Mail: hasbullah.thabrany@ui.ac.id

**Keywords:** cardiovascular diseases, smoking, Indonesia

## Abstract

The main objective of this study is to present the prevalence of Cardio Vascular Diseases (CVDs) defined as been diagnosed or having symptoms of Coronary Heart Disease, Arrhytmia, or Heart Failure. The main risk factor analyzed is smoking behavior. The data used for this study was from Basic Health Survey of 2007, a National baseline data collected every three years which consist of more than one million samples representing 33 provinces in Indonesia. Information on socio-demographic characteristics, history of CVDs and smoking behavior were collected by highly-trained interviewers using a questionnaire which had been tested. A sub-sample of the survey consisting of 100,009 males aged 45 years and over was analyzed. Crude and adjusted odds ratio (OR) were analyzed using logistic regressions to estimate the prevalence of CVDs by smoking behavior and socio-demographic characteristics. Overall, 86.8% respondents reported that they had never been diagnosed as having CVDs or having any symptom of CVDs.; while 2.1% respondents reported that they had been diagnosed by a health professional (a doctor or a nurse) of having CVDs. The interviewers also identified three signs and symptoms of CVDs for all respondents if they reported of never been diagnosed CVDs. Among all respondents 2.3% had symptoms of coronary heart disease, 4.9% had symptoms of arrhytmia, and 3.9% had symptoms of heart failure. The prevalence of CVDs was significantly higher in former smokers (OR = 2.03), and duration of smoking for more than 20 years. The prevalence of CVDs was significantly higher among older groups. Old males who lived in Sulawesi island had higher probability of having CVDs (OR = 1.67). The lower prevalence of CVDs seemed to have associated with higher among Senior High School Graduate compared to those who Never Schooling (OR = 0.8). Since population of Indonesia is relatively young, the future of health care costs of Indonesia would be high due to high prevalence of smoking among males population. This finding suggests that Indonesia should ratify Framework Convention on Tobacco Control ans start impelementing measures to control tobacco uses in order to reduce public health and economic consequences of smoking in the future.

## 1. Introduction

Series of National Household Surveys in Indonesia indicate increasing rank of CVDs as the main cause of death. It ranked 11th in 1972, ranked 3rd in 1986 and rapidly increased to the 1st rank in 1992, 1995, and 2001 respectively [1]. According to Basic Health Survey or BHS 2007, the prevalence of smoking among Indonesian aged ≥15 years is 29.2% (23.7% regular and 5.5% occasional smokers, 46.8% among males and 3% among females) [2]. Various studies indicate that smoking has been a significant risk factor for coronary heart diseases (CHDs). Smoking accelerates coronary plaque development and it is a strong risk factor for myocardial infarction. The Framingham study assigned 2 (two) risk points for smoking that seems appropriate for patients who smoked about one pack of cigarettes a day. Those who smoked more than one pack of cigarette a day were extremely at high risk for premature CHDs [3]. A case-control study found that the OR for Myocard Infark (MI) showed a strong dose-response relationship, with an odd ratio (OR) of 2.47 for those who smoked 1 to 5 cigarettes/sticks per day and with an OR 74.6 for those who smoked more than 40 cigarettes per day as compared to nonsmokers. Cigarettes also have been associated with sudden cardiac death of all types with a relative risk (RR) of 2.5 compared with nonsmokers [4].

Smoking reduces the desire to do sport and increases the chance of suffering from influenza and cough or prolongs duration to heal [5]. Lack of sport or physical inactivity is another risk factor for cardiovascular diseases. In the USA, smoking was the first leading cause of death in 2000, which is followed by physical inactivity and poor diet [6]. Although there have been decreasing trend of smoking in developed countries, in developing countries, the number of smokers has been increasing [7]. In Indonesia, the prevalence of smoking among males aged 10 years or older increased from 46.4% in 1980 to 52.9% in 1986. It decreased slightly to 51.3% in 1995 but increased again to 54.5% in 2001 [8–10].

According to BHS 2007, Sulawesi has been found not only as a territory of high prevalence of tobacco smoking, but it is also as the territory where the majority of smokers start smoking in a relatively younger age. Three of six provinces of Sulawesi Island have higher smoking prevalence than the national average. The (male and female) smoking prevalence in these provinces: North Sulawesi, Central Sulawesi and Gorontalo were 30.3%, 30.7% and 32.6% respectively; while the national prevalence of smoking was 29.2%. Furthermore, the proportion of smokers who have started smoking regularly since aged 10–14 years in North Sulawesi, Central Sulawesi and Gorontalo were 10.4%, 10% and 12.9% respectively. While nationally, the proportion of smokers who have started smoking since the same age was 9.6% [2]. It means that at the age of 50 years old, smokers in Sulawesi have longer duration of smoking than smokers in other provinces.

To reduce the risks of smoking, the World Health Organization (WHO) has encouraged it’s member countries to ratify the Framework Convention on Tobacco Control (FCTC) [11]. Unfortunately, until the end of 2010, the Government of Republic of Indonesia has not ratified it. One of the reasons was the government officials perceived of lack of local evidences on the health consequences of smoking in Indonesia. Therefore, domestic studies on the risks of smoking on health consequences among Indonesian population are needed.

The impetus for domestic studies in Indonesia becomes more important as the majority of cigarettes consumed by Indonesian are kreteks. While some policy makers argue that the kreteks (the Indonesian native cloved cigarettes) have no health consequence, a study uncovers that kreteks have been proven to be more cariogenic than cigarettes without clove [12]. Since the associations between kreteks and CVDs have not been proven, this study tried to examine that relationship. One of the research questions is: “is there any evidence that kreteks smokers have higher risks of CVDs than those of non-kretek smokers?” The ideal research to answer the question is a cohort study involving more than 20,000 adult males followed for a 5-year annual follow up, such had been performed in Shanghai—China [13]. In Indonesia, such study has never been done and it is unlikely in the near future; while the kretek-CVD relationship is urgently needed for policy advocy. We believe that the BHS data could provide valid and reliable estimates of OR for policy decision. To support policy advice, in this study, we examined OR of smoking behaviors and socio-demographic determinants to CVDs using the BHS 2007 data.

## 2. Method

The samples for the BHS 2007 were drawn from the National sampling frame developed by the Central Bureau of Statistic. The survey collected data from more than 200,000 households consisting of about 1.1 million household members. The survey was conducted by the National Institute of Health Research and Development, the Ministry of Health, Republic of Indonesia. The data collection was fielded from August 2007 to August 2008 using a set of pre-test questionnaires. Various socio-demographic, economics, and behavioral variables—including smoking and history of diseases were collected. Interviewers were health professionals, who were highly trained to conduct the interviews and to observe certain signs and symptoms of CVDs.

Previous surveys indicated that more than 50% of Indonesian males aged 15+ years were smokers (compared to less than 5% of females). The selection of males age (45+ years) for this study was designed to allow analyses on the effects of long duration of smoking (of at least 20 years) on CVDs. A previous longitudinal study by Yan *et al.* (1996) had proven the effects of smoking on cardiovascular morbidity and mortality in China, for people 45+ years of age [13].

Although 106,454 male respondents aged 45+ years were selected, analysis was restricted to the sub-sample comprised of 100,009 respondents, while 6,445 respondents were dropped because no information on smoking behavior. The determination of CVDs as the dependent variable was made on two methods: First, the prevalence of CVDs was determined by asking respondents whether they have ever been diagnosed by a health professional (a medical doctor or a nurse) of suffering a CVD. The “yes” answer leads to classification of “diagnosed CVD”. Second, respondents who answered “no”, were further asked and observed of whether they had ever experienced one of the following signs and symptoms:

1. If a respondent reported that he had experienced chest pain or discomfort (angina), felt heavy or feeling of someone was squeezing his heart, felt pain under his breast bone (sternum), in his neck, arms, stomach, or upper back muscles then he was classified as having symptoms of coronary heart disease. The respondents were securely understood that the pain usually occurs with certain activities or emotion. Other symptoms included shortness of breath and fatigue with relatively mild activities (exertion) [14].
2. If a respondent reported that he experienced disorders of regular cardiac rhythmic (heart beat), the heart beat was too fast, too slowly, or erratically/irregular then he was classified as having symptoms of arryhtmia [15].
3. If a respondent reported that he experienced: shortness of breath, frequent coughing, especially when he was lying down, swollen feet, ankles, or legs, having abdominal pain, fatigue, dizziness or fainting then he was classified as having symptoms of heart failure [16].

If a respondent reported or was found of having at least one of the above three conditions, then the respondent was classified as having ‘undiagnosed CVDs’ in this study. The determination of ‘undiagnosed CVDs’ or having symptoms of CVD was made to accommodate the fact that not all Indonesian have access to a health professional or a health facility due to financial, geographical, or educational barrier.

The relationship of CVDs with the main variable of smoking behavior was analyzed. Other determinants or risks factors for CVDs that were assesed are: (1) urban-rural residence (dummy); (2) territorial/islands of residence (Java, Sumatra, Kalimantan, Sulawesi, Bali and Nusa Tenggara, and Maluku and Papua); (3) age (year); (4) Level of education (Never Schooling, some Primary School, graduate Primary School, Junior High Schoold Graduate and Senior High School Graduate); (5) level of expenditure (by income quintiles); (6) type of cigarettes (non kretek only, mixture of non kretek and kretek, and kretek only); (7) the number of sticks smoked per day, and (8) duration of smoking (years).

The smoking behavior as the main independent variable was classified into for groups as follow:

Never smoke—“I have never tried smoking”
Former smokers —“I have quit smoking”.
Occasional smokers—“I smoke, but not every day”.
Regular smokers —“I smoke at least one cigarette a day”.

For regular smokers, we calculated the duration of smoking by substracting the current age minus the age of starting smoking. The total number of respondents for each pair of variables may vary slightly due to missing data.

Data base was set up and analyzed using SPSS version 15. The chi-square (χ^2^) test was used to compare proportions of CVDs by age groups and smoking status as shown in Table 1. The adjusted odds ratios were estimated using logistic regression analyses as shown in Table 2. Duration of smoking, average number of cigarette smoked per day and type of cigarette smoked were analyzed to estimates the risks on CVDs.

## 3. Results

The proportion of smokers among males aged ≥45 years respondents was 72%, which is consistent with previous surveys. However, as much as 63% of them were occasional smokers. Overall, 2.1% males 45+ years had been diagnosed by a health professional as suffering a CVD. Of all respondents: 2.3% had symptoms of Coronary Heart Disease (CHD), 4.9% had symptoms of Arrhytmia, 3.9% had symptoms of Heart Failure. Totally, the prevalence of CVDs was 13.2%. The proportion of respondents with diagnosed CVD, having sympotoms of CHD, Arrhytmia, and Heart Failure increased with age (Pearson r = 0.95, P < 0.005). Proportion of respondents with diagnosed CVDs were relatively lower than those of undiagnosed (having symptoms) of CVDs at any age—reflecting poor access to health facilities. Former smokers were more likely to have either diagnosed CVD, undiagnosed CHD and heart failure; while regular smokers were more likely to have arrhythmia (Table 1).

Table 1 shows that age group was associated with higher risks of CVDs significantly. The table also shows that a number of 62,659 (63%) of total respondents were regular smokers while 2,159 (2%) weere occasional smokers. These prevalences were higher than the prevalence of regular smokers among malse aged 10+ years which was 45.8% for occasional smokers which was 9.9% [2]. A significantly higher risk CVD was associated with former smokers. Among regular smokers, the CVD prevalence among those who smoked for ≥20 years was higher than that of those who smoked for less than 20 years. Our in depth analysis found that 99.9% of former smokers smoked for more than 20 years.

The “mixture” of non kretek and kretek had highest risk of CVD. A significantly lower risk of CVDs was associated with increasing level of education (Table 2). There was relatively no difference on CVDs prevalence among rural and urban residents. Higher prevalence of CVDs (combined diagnosed and undiagnosed/having symptoms) was higher among respondents who lived in Sulawesi Island (territory) compared to those who lived in Maluku and Papua with OR of 1.67 (CI: 1.45–1.91). When adjusted the level of expenditure was not significantly associated with a higher risk for CVD.

## 4. Discussion

In this study, we examined the prevalence of CVDs in a large survey of Indonesian males aged 45 years and over, a sub-sample of Basic Health Survey (BHS) 2007. We found that 2.1% respondents had been diagnosed by a health professional as suffering from at least a CVD (for further discussion we named it diagnosed CVDs). We also found that, among those who had never been diagnosed with a CVD, there a higher percentage of respondents had experienced CVD symptoms such as CHD (2.3%), arrhythmia (4.9%) and heart failure (3.9%). We classified the later cases as undiagnosed CVDs. The assessment of symptoms of CVDs was based on a pre survey assumption that access to a health professional or a health facility has been difficult for many Indonesians due to lack of insurance, cultural barriers, and geographical barriers. Although it was done by a well trained interviewers, there were potential biases (over or under-diagnosed due to recall or subjective symptoms). However, this assessment is essential for policy and planning of health coverage. By assessing undiagnosed CVDs, the author could estimate potential needs and demands for services of CVDs. The higher prevalence of undiagnosed CVDs may indicate that there had been the needs for health coverage as of 2007, about 120 million Indonesians had no health insurance. This year, the government declare its political commitment to expand health coverage to achieve universal health coverage by 2014, an ambitious program, yet it is supported by evidences found by this study.

In order to simplify the assessment the authors combined analysis on the prevalence of diagnosed and undiagnosed CVDs, although there may have some biases. When compared to the never-smokers as the reference, current smokers who had smoked for <20 years have OR = 1.43 (CI: 1.17–1.76) to suffer from diagnosed or undiagnosed CVDs, while those who had smoked ≥20 years have OR = 1.26 (CI: 1.18–1.35) to suffer the diseases. On the other hand former smokers had twice more likely of having CVDs compared to non smokers (see Table 2). Former smokers (who stopped smoking by the survey time were probably stopped smoking after long period of smoking and having some symptoms or had been diagnosed for a tobacco related diseases). Thus, this study confirms that smoking clearly increased the risks of having CVDs for Indonesians.

This study identifies a little bit higher OR on duration of smoking for less than 20 years as compared to those who smoked for more than 20 years, but the different was not so large. Both had higher probability of having CVDs compared to non smokers. This finding may be related to the fact that the number of unidentified or “unknown duration by respondents” among current smokers is relatively high. Secondly, some of smokers for more than 20 years were already grouped in former smokers. Detailed analysis found that 99.9% of formers smokers actually had smoked for >20 years. A further analysis uncovered that the majority of those who smoked for >20 years had quit smoking as soon as they had been diagnosed as having or experienced symptoms of CVD. So, the authors firmly conclude that the higher prevalence of CVDs was associated with longer duration of smoking.

A relatively small difference on the prevalence of CVD between those who smoked <20 sticks a day and those who smoked ≥20 sticks a day might be attributable to recall bias or marginal cut off that might be actually at 10–15 sticks per day rather than 20 sticks. The facts that former smokers have the highest CVD prevalence, 87.9% of former smokers smoked between 1–12 cigarette per day, and that 99.9% of them were smoking for more than 20 years. Based on these finding the authors in favor of view that duration of smoking have a greater effect on CVDs than the number of sticks per day.

Comparing the prevalence of CVDs among non kreteks, mix, and kreteks only smokers, the authors found that prevalence of CVD was higher among those who smoked non kreteks or mixture of non kreteks and kreteks rather than among those who smoked kreteks only. This finding may not good for policy advice since some policy makers already raised their view of kreteks (with clove in it) had less health impact compared to non-kreteks. However, policy advisers must ensure the policy makers that there is no way to guarantee that Indonesian smokers would not smoke non kreteks.

With the reference of respondents who never schooling, respondents who had at least 12 years of education (Senior High School graduate) had less likely to suffer from a CVD with OR as low as 0.8 (0.72–0.88). From public health point of view, two findings of this study (higher prevalence of CVDs associated with longer duration of smoking and lower prevalence of CVDs associated with longer education might be used as a weapon for tobacco control activists and to push the Indonesian Government to ratify WHO FCTC. Indonesia should implement effective youth smoking prevention programs including total ban of tobacco advertising and promotion, and prohibition of cigarette sale to minors. Well planned and financially supported education on “Tobacco or Health” should also be given as part of curricula in at least 12 years of basic education.

Although the causal relationship between smoking epidemic and it’s public cardiovascular health consequences in our study is not as strong as an experimental or cohort study, some findings are consistent with previous cohort studies from other countries. As described previously, many Indonesian policy makers are still in doubt to ratify FCTC due to inadequate scientific evidences from domestic studies, this study could assist anti smoking activists to convince the policy and legal makers. Previously, Indonesian tobacco control activists and advocates have no strong weapon to fight such statement. The endeavors of anti tobacco activists and advocates to ratify FCTC have been failed so far, which in some part, due to a strong resistance by National Parliament [17].

The authors realize that there are some limitations of this study. For example, there were unidentified cases that might resulted in underestimate OR for some groups. The ideal study should be a cohort study, but it needs long time to produce a result while at the same times mortality and morbidity from the existing smoking behavior will be high. Furthermore, this study does not estimate the public health burden of tobacco epidemic in a comprehensive way, such as it’s economic consequences. Another study based on a household survey found that the cost of tobacco related diseases in Indonesia has been more than three times higher than education and health costs [18].

By September 22nd, 2009, Indonesian clove cigarette (*rokok kretek*) such as A Mild, Bentoel Class Mild, Djarum Black, Djarum Coklat, Bentoel Star Mild, Djarum Black Cappuccino, Djarum LA Lights, GG Surya Slim 16 White Edition, Marlboro Filter Kretek Mix 9, Sampoerna A Mild, Sampoerna Dji Sam Soe 234 Magnum, Sukun Orange Kretek, and Tali Jagat Kretek (referred as flavored cigarette products) has been banned by US Food Drug and Administration to enter US market [19]. Kartono Mohammad, an Indonesian prominent tobacco control advocate stated: “Unless Indonesian Government stand on the side of the Indonesian public, this rule of the US Government might even give heavier public health burden to the people of Indonesia”. He also predicted that trans-national tobacco industries in Indonesia will sell out those rejected products into Indonesian domestic market— with cheaper prices—in order to reduce their dead lost. Lower income Indonesians will be more likely to buy those lower prices cigarette now but costs more to them in few decades ahead. While this situation become public health disaster for Indonesians, in the contrary, the economic benefit of tobacco industries in Indonesia will be enjoyed in a larger part by foreigner, since some tobacco industries in Indonesia are now taken over by Foreign Companies (Philip Morris and British American Tobacco) [20].

Trends in tobacco use among Indonesian are increasing. The total number of cigarette consumed by Indonesian in the year 1990, 1997 and 2000 were 145.7, 202 and 229 billion sticks respectively [21]. By consuming 182 billion sticks of cigarette in the year 2002, Indonesia ranked 5th among top 5 cigarette-consuming countries in the world. By consuming 239 billion sticks of cigarette in the year 2007, Indonesia is still in that position [22]. Indonesia is one of countries with severe under-nutrition problem. In the year 2004 more than 5 million (28.47%) children under five years were undernourished [23]. It is far from justice that millions of children are suffering from under-nutrition but millions of Indonesian fathers spend their money on cigarettes more than on meat, eggs or milk for their children.

## 5. Conclusions

Our study has shown that the doubt about the impact of cigarettes on cardio-vascular diseases in Indonesia is irrational. This study found that the longer the duration of smoking the higher the probability of the smokers to have CVDs. Those who smoked cigarettes for more than 20 years have about twice the risks for CVDs compared to those who never smoked. Since the difference on the impact of smoking kreteks and non-kreteks cigarette to Indonesian public cardiovascular health remained unclear from this study, we recommend to conduct cohort prospective studies on mortality and morbidity in relation to cigarette smoking in Indonesia. Such studies are necessary, not only to answer questions on public health consequences of kretek cigarette smoking, but also to answer questions on prevalence of smoking-related cancers. The authors strongly recommend the Government of Republic of Indonesia to open their eyes to see the future burden of health care costs, if the smoking epidemic remains uncontrolled. The authors also suggest Indonesian tobacco control activists and advocates to strengthen public pressures to the Indonesian government to access FCTC.

## Figures and Tables

**Table 1.** Distribution of the prevelance of CVDs by age and smoking status among Indonesian male respondents age 45+ years, 2007.

		Total respondents	Diagnosed CVDs	Symptom of CVD (undiagnosed CVDs)			Sub total symptom of CVDs	Prevalence of all CVDs
				Coronary HD	Arrhythmia	Heart Failure		
Unit		100%	(%)	(%)	(%)	(%)	(%)	(%)
**Total**		**100,009**	**2.1**	**2.3**	**4.9**	**3.9**	**11.1**	**13.2**
**Age**^a^								
	45–54	47,429	1.5	1.7	4.2	2.6	8.5	**10.0**
	55–64	28,252	2.4	2.3	5.2	3.9	11.4	**13.8**
	65–74	16,749	3.0	3.1	6.0	6.1	15.2	**18.2**
	≥75	7,579	2.9	3.6	6.0	7.8	17.4	**20.3**
**Smoking status**^a^								
	Regular Smokers	62,659	1.2	2.3	5.1	3.7	11.1	**12.3**
	Occasional Smokers	2,159	0.8	2.2	6.8	5.4	14.4	**15.2**
	Former Smokers	12,545	5.8	3.2	5.9	6.1	15.2	**21**
	Never Smoked	22,646	2.5	1.5	3.8	3.2	8.5	**11**

a *P* < 0.005 (χ^2^ test).

**Table 2.** Distribution of Prevalences and Odd Ratios for CVDs by Smoking Behavior and Socio-Demographic Variables among Indonesian Males Aged 45+ years, 2007.

	**No.**	**CVDs (*diagnosed***&***by symptoms*) (%)**	**Adjusted OR**
**Duration of Smoking**			
Never	22,646	11.0	1^a^
Former Smokers	12,546	21.0	2.03 (1.87–2.20)^b^
Smoked <20 years	1,660	14.0	1.43 (1.17–1.76)
Smoked ≥20 years	37,075	13.0	1.26 (1.18–1.35)
Unidentified	26,082	11.6	1.01 (0.94–1.09)

**No. of cigarette smoked Per day (sticks)**			
Never	22,646	11.0	1 ^a^
Former Smokers	12,546	21.0	2.03 (1.87–2.20) ^b^
Smoke <20 sticks	62,658	12.4	1.16 (1.09–1. 24)
Smoke ≥20 sticks	2,159	15.1	1.39 (1.19–1.63)

**Types of cigarette smoked**			
Never smoked	22,646	11.0	1^a^
Former Smokers	12,546	21.0	2.03 (1.87–2.20)^b^
Non Kreteks	12,973	14.3	1.16 (1.06–1.27)
Mix kreteks-non kreteks	13,797	14.7	1.37 (1.25–1.49)
Kreteks only	36,970	11.1	1.09 (1.02–1.17)

**Residences**			
Urban	41,171	12.3	1^a^
Rural	58,838	13.8	1 (0.94–1.07)^b^

**Territories**			
Maluku and Papua	1,755	11.9	1^a^
Java	64,453	12.6	0.95 (0.84–1.09)^b^
Sumatra	17,659	12.5	0.99 (0.87–1.13)
Kalimantan	4,783	14.2	1.17(1.01–1.36)
Sulawesi	6,151	19.7	1.67 (1.45–1.91)
Bali & Nusa Tenggara	5,208	15.0	1.21 (1.03–1.41)

**Age groups (Years)**			
45–54	47,429	10.0	1 ^a^
55–64	28,252	13.8	1.38 (1.29–1.46) ^b^
65–74	16,749	18.1	1.81 (1.69–1.93)
≥75	7,579	20.2	1.98 (1.82–2.16)

**Level of education**			
Never schooling	14,215	15.9	1^a^
Some primary school (PS)	26,252	14.8	1.04 (0.96–1.12)^b^
PS graduate	30,712	13.1	0.95 (0.88–1.03)
Junior HS graduate	10,091	11.0	0.83 (0.74–0.91)
Senior HS graduate	18,486	10.4	0.80 (0.72–0.88)

**Household Expenditures**			
Lowest Quintile	20,877	14.1	1^a^
Middle Low	20,539	13.8	0.99 (0.92–1.07)^b^
Middle	19,981	13.2	0,96 (0.89–1.04)
Middle High	19,744	12.7	0.94 (0.87–1.01)
Highest Quintile	18,672	12.0	0.94 (0.87–1.02)

a Reference class;

b Figures in parentheses are 95% confidence intervals (estimates obtained using logistic regression).
